# A rare case of gastrointestinal amyloidosis secondary to myeloma with predominant jejunal involvement

**DOI:** 10.1093/jscr/rjab639

**Published:** 2022-01-26

**Authors:** Manoj Anandan, Lei Ying, Benjamin Macisaac, Alexander Kilner, Robyn Laurie, Uvarasen K Naidoo

## Abstract

Amyloidosis is a condition identified by the accumulation of abnormal proteins in various tissues and organs that eventually lead to impaired function. Systemic amyloidosis with gastrointestinal (GI) tract involvement is more common than localized GI amyloidosis, whereas predominant jejunal involvement is even more uncommon. We report a rare case of systemic amyloidosis with predominant jejunal involvement in a 76-year-old female who presented with lower abdominal bloating and lethargy.

## INTRODUCTION

Amyloidosis is a rare disorder defined by the extracellular deposition of abnormal, insoluble and fibrillar proteins in various tissues and organs, which eventually cause distortion of tissue architecture and function. It can be broadly classified into systemic or localized amyloidosis [1]. Systemic amyloidosis can be primary, secondary, hereditary, haemodialysis related and senile, which contribute to the majority of cases with at least 50% involving the gastrointestinal (GI) tract [2]. Localised amyloidosis involving the GI tract that produces symptoms is rare [2]. The clinical features of amyloidosis may be non-specific such as fatigue, light-headedness, anorexia and weight loss or specific with GI symptoms such as epigastric pain, vomiting, diarrhoea, bloating and GI bleeding [1, 3]. We present a case of GI amyloidosis secondary to myeloma predominantly affecting the jejunum.

## CASE REPORT

**Figure 1:**
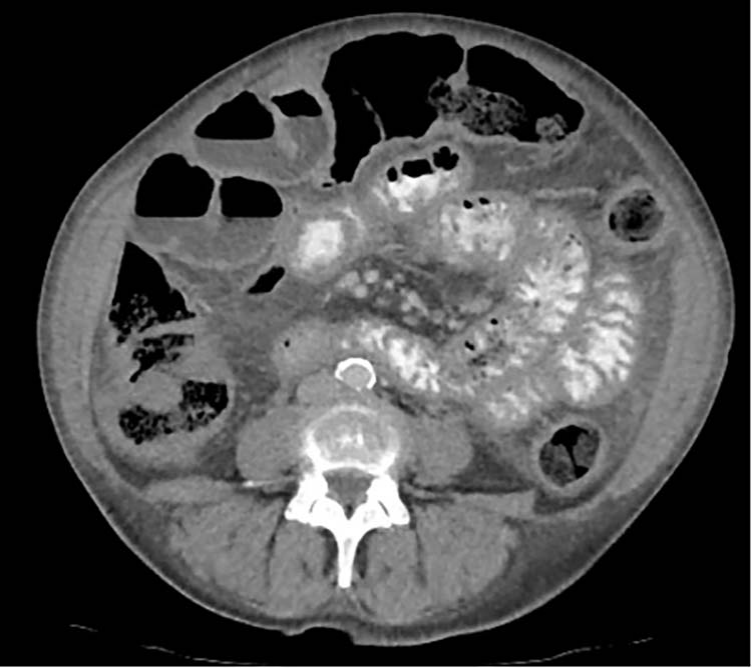
Axial CT image displaying diffuse thickening of the small and large bowel with dilatation of the small bowel.

**Figure 2:**
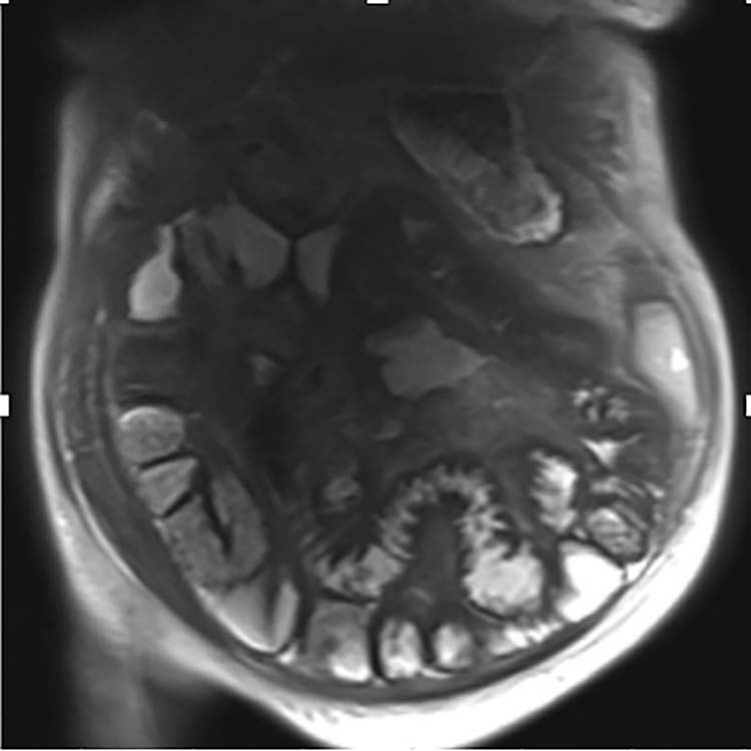
Coronal magnetic resonance image of abnormal thick-walled loops of jejunum and proximal ileum without focal stricture.

**Figure 3:**
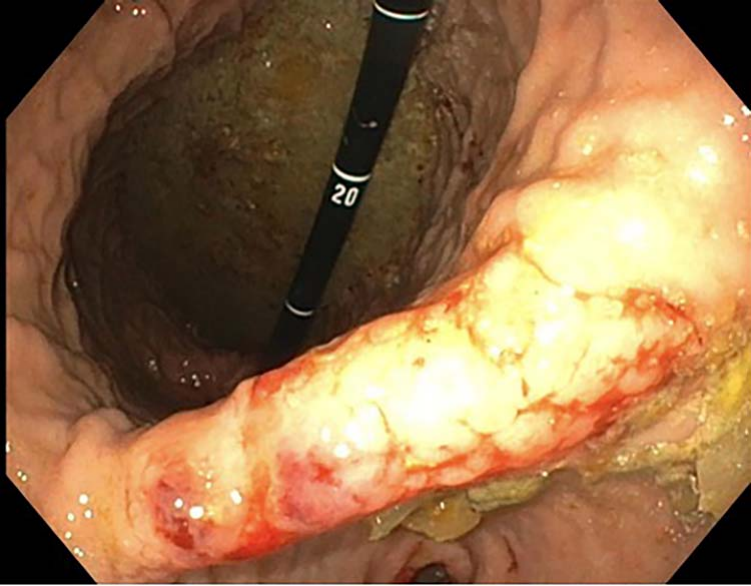
Endoscopic retroflexed view of the stomach showing gastritis, patchy telangiectasia and sloughy mucosa at the incisura.

**Figure 4:**
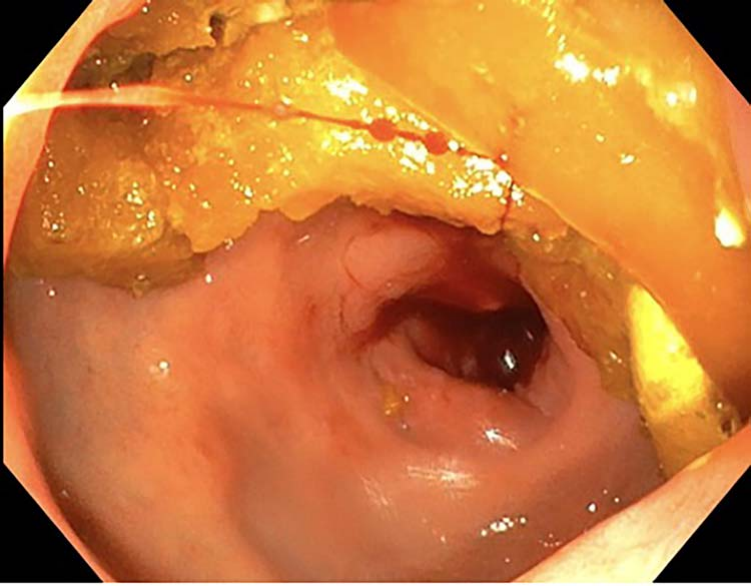
Endoscopy showing duodenitis with stricture at the second part of duodenum (D2).

**Figure 5:**
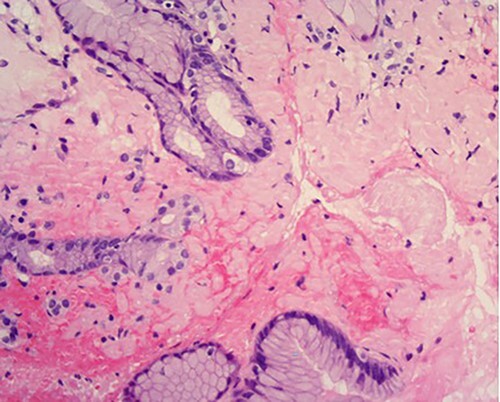
High power of proximal jejunum biopsy showing extensive submucosal deposition of pink amorphous material (H&E ×20).

**Figure 6:**
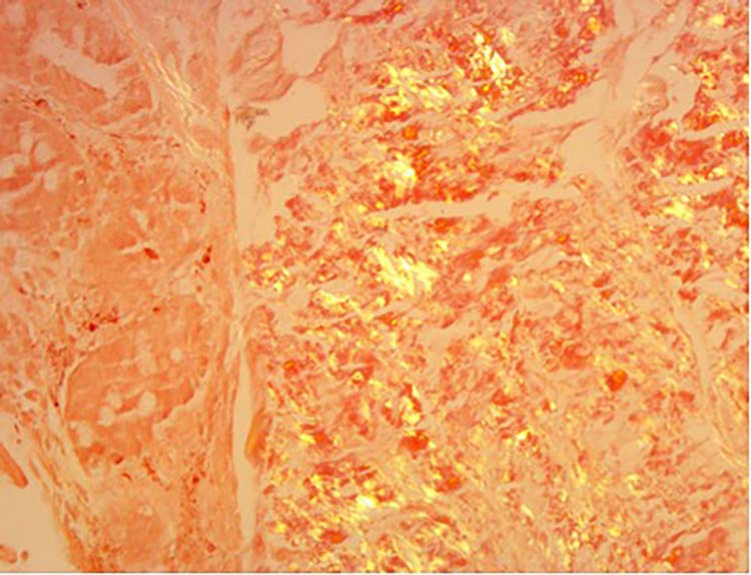
Amyloid stain on proximal jejunum biopsy as seen under polarized light showing apple-green birefringence (Congo Red ×20).

A 76-year-old lady presented to the outpatient clinic with a history of lower abdominal bloating and lethargy for 1 week. The patient denied abdominal pain, nausea, altered bowel habits or GI bleeding. Her past medical history included gastroesophageal reflux disease, ulcerative colitis and a previous hysterectomy. Physical examination exhibited pallor of the conjunctiva, distended abdomen with visible peristalsis on inspection but soft, non-tender on palpation and absence of ascites. Laboratory testing found normocytic normochromic anaemia, thrombocytosis and a positive myeloma screen with elevated kappa to lambda free light chain ratio despite normal renal and liver function. An ultrasound of the abdomen was unremarkable. Computed tomography (CT) with IV contrast of the abdomen revealed diffuse thickening of the small and large bowel with the small bowel dilated throughout. Magnetic resonance imaging of the small bowel showed multiple abnormally thickened loops of the jejunum and proximal ileum with a maximal wall thickness of 0.9 cm (Figs 1 and 2). Gastroscopy revealed severe distal oesophagitis, the stomach contained patchy telangiectasia and gastritis with sloughy mucosa at the incisura in addition to duodenitis with stricture at the second part of duodenum (D2) (Figs 3 and 4). Histopathology from the biopsies of the gastric incisura, D2 and the proximal jejunum displayed reactive changes with intramucosal haemorrhage and extensive deposition of pink amorphous, eosinophilic material on haematoxylin & eosin (H&E) stain. The deposits were predominantly seen in the proximal jejunum with a positive Congo red stain showing apple-green birefringence under polarized light (Figs 5 and 6). The patient was referred to Haematology, and a bone marrow aspirate and trephine biopsy revealed proliferation of 15% mature plasma cells consistent with the diagnosis of myeloma.

## DISCUSSION

Amyloidosis is a disease that affects multiple tissues and organs by causing accumulation of abnormal and misfolded extracellular proteins [4]. Immunoglobulin derived light chain (AL amyloidosis), which is commonly associated with plasma cell dyscrasias, and amyloid A (AA amyloidosis), which is also an acute phase reactant that is commonly seen in chronic inflammatory diseases, are among the commonest precursors of amyloidosis [5]. On the other hand, haemodialysis-related b-2 microglobulin (B2M) due to decreased renal clearance of B2M is commonly seen in patients who undergo haemodialysis for end-stage renal failure and apolipoprotein E (Alzheimer’s related) [5].

Primary amyloidosis is associated with multiple myeloma in 15–20% of cases, whereas primary amyloidosis of the GI tract on the other hand is rare with only 1% having had biopsy-proven symptomatic gastric amyloidosis based on a case series of 769 patients with primary systemic amyloidosis [6]. The highest diagnostic yield from biopsy specimens was found to be the duodenum (50%) followed by stomach (44%), colon (32%), oesophagus (12%) and rectum (8%) [7]. Amyloidosis commonly affects older people with a mean age of 63, the incidence being higher amongst males [1]. Studies have shown that 98% of patients with amyloidosis have subclinical GI disease; however, only 50% with GI involvement will be symptomatic [8].

The pathogenesis of GI involvement in amyloidosis can be broadly divided into mucosal or neuromuscular infiltration. The layers involved in mucosal infiltration depends on the type of amyloid deposition as AL amyloidosis can involve muscularis mucosa, submucosa and muscularis propria forming protrusions that lead to symptoms of bowel obstruction, whereas AA amyloidosis mainly affects the mucosa which leads to erosions and manifest as diarrhoea and malabsorption [1]. The deposition of amyloid protein in the neuromuscular layer can affect the intrinsic nerve plexus (submucosal or myenteric), which leads to abnormal peristalsis and dysmotility [1]. Although cutaneous, renal and cardiac involvement are common, GI involvement occurs in only 3–8% of AL amyloidosis [7].

The investigation for AL amyloidosis should include testing of serum and urine for monoclonal light chains by immunoelectrophoresis and immunofixation followed by a bone marrow aspirate for quantification of the number of plasma cells as well as to establish presence of monoclonal chains. Endoscopic examinations and biopsy is beneficial; however, some patients may require push enteroscopy or capsule endoscopy to detect lesions that are limited to the jejunum [3]. The gold standard for diagnosing amyloidosis remains visualization of the characteristic green birefringence under polarized light following Congo red staining of the tissue biopsy [3].

The management of amyloidosis depends on the amyloid subtype and the extent of involvement. AL amyloidosis commonly requires chemotherapy and in certain cases haematopoietic stem cell transplantation to remove plasma cell clones. The treatment of AA amyloidosis depends on managing the underlying inflammatory condition to diminish disease progression such as the use of anti-tumour necrosis factor in Crohn’s disease [9]. The amyloid subtype and extent of tissue involvement both determine the prognosis of amyloidosis. Unfortunately, AL amyloidosis with GI involvement has a poorer prognosis with a median survival of 6.3 years. This is based on the complications that arise such as malnutrition, intestinal bleeding, pseudo-obstruction secondary to intestinal dysmotility and rarely perforation [8]. Supportive measures for GI symptoms remain the mainstay of treatment; however, surgical intervention is necessary in cases of fatal GI haemorrhage, obstruction or perforation [3, 5].
